# Acute Mastoiditis Complicated with Concomitant Bezold's Abscess and Lateral Sinus Thrombosis

**DOI:** 10.1155/2018/8702532

**Published:** 2018-03-20

**Authors:** Aziz Mustafa, Burhan Toçi, Hajdin Thaçi, Bujar Gjikolli, Nehat Baftiu

**Affiliations:** ^1^ENT Clinic, University Clinical Center of Kosova, Prishtina, Kosovo; ^2^Radiology Clinic, University Clinical Center of Kosova, Prishtina, Kosovo; ^3^Anesthesiology/Reanimation Clinic, University Clinical Center of Kosova, Prishtina, Kosovo

## Abstract

Bezold's abscess is a very rare extracranial complication of acute mastoiditis. Lateral sinus thrombosis is another intracranial complication of acute mastoiditis that can occur, but there are only few reports of concomitant ispilateral Bezold's abscess and lateral sinus thrombosis with favorable outcome. We diagnosed and treated successfully a 14-year-old girl suffering from *Streptococcus pneumoniae* acute mastoiditis complicated with Bezold's abscess and lateral sinus thrombosis. Surgical treatment included myringotomy, cortical mastoidectomy, and Bezold's abscess incision and drainage. During the course of treatment, we concluded that lateral sinus thrombosis was not caused from mastoiditis by direct spread but from pressure on internal jugular vein caused from Bezold's abscess.

## 1. Introduction

Acute otitis media (AOM) is a frequent infection in childhood, mostly with viral etiology, but bacterial AOM and acute mastoiditis (AM) can be complicated with life-threatening either extracranial complication (EEC) or intracranial complication (ICC). Named after German otologist Friedrich von Bezold (1842–1908), who in 1881 was the first author to describe neck abscess along the sternocleidomastoid muscle (SCM) [1], Bezold's abscess (BA) is a severe and very rare ECC of suppurative AOM or cholesteatomatous chronic otitis media (CCOM). In cases of severe bacterial infection of the tip of mastoid, the suppurative content from the mastoid air cells can descent along the upper insertion of sternocleidomastoid muscle and cause collection of the pus between muscle and its fascia. If not treated in time and properly, content from BA can descent to mediastinum, causing acute mediastinitis, another complication with mortality of 70% [2–6]. Comparing with BA, the lateral sinus thrombosis (LST) is an another life-threatening ICC of ear infections, which can be seen more often in combination with other ICCs, but there are only few reports on concomitant ECC/ICC (BA/LST) complications arising from acute mastoiditis [2, 7]. Mainstay in diagnostic of ECC and ICC is clinical imaging, computed tomography (CT), or magnetic resonance imaging (MRI) [8]. Besides the intravenously administered antibiotics, the surgical treatment (mastoidectomy, myringotomy, and abscess drainage) is mandatory for proper management and prevention of sequels. In cases of sinus thrombosis, anticoagulation therapy is not definitively associated with improved outcomes and warrants further research [9–11].

This article describes a favorable outcome case report of concomitant ECC/ICC, BA/LST and discusses management of these complications.

## 2. Case Presentation

**Figure 1:**
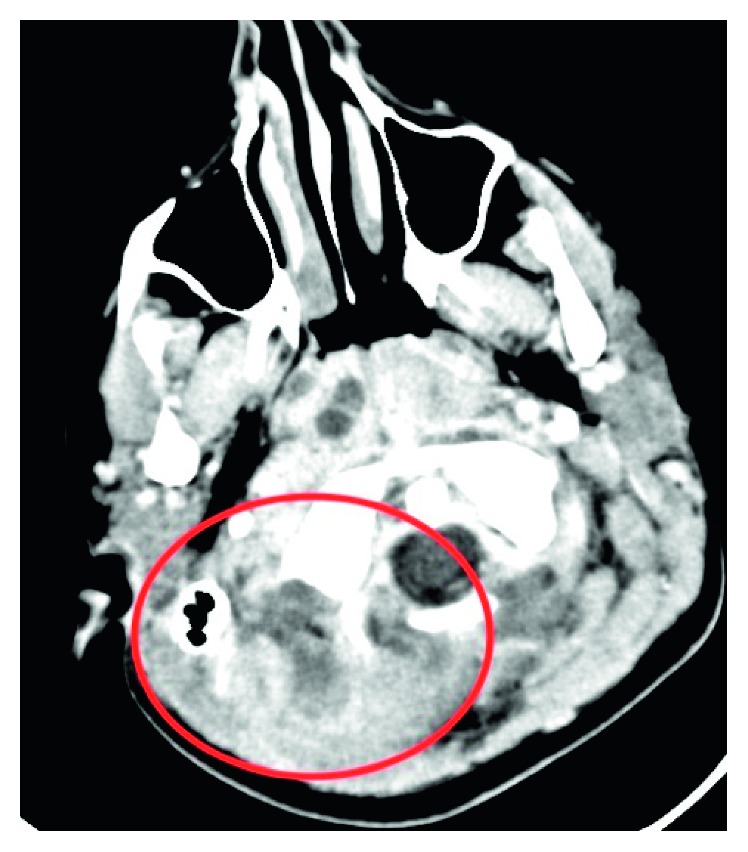
Irregular hypodensity below the right mastoid and right half of the occipital bone surrounded with postcontrast (red circle) increase of density represent the abscess formation.

**Figure 2:**
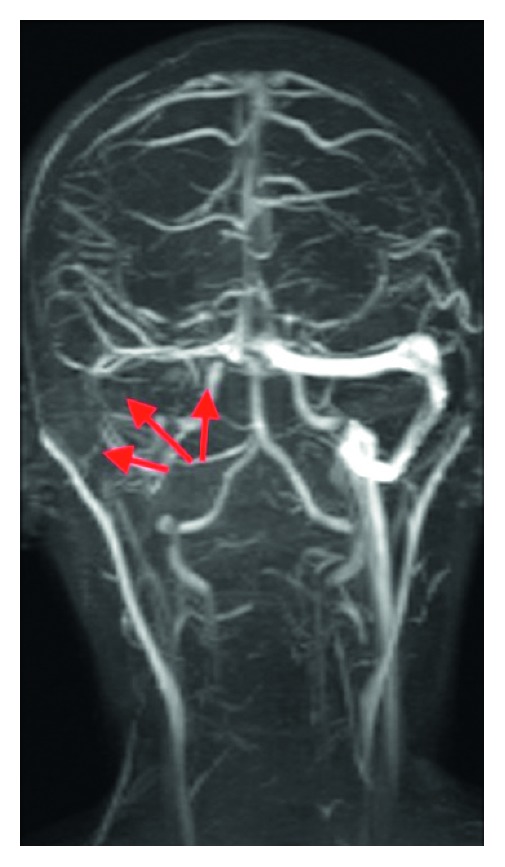
Coronal MR venography 3 weeks after operation shows missing right transverse and sigmoid venous sinus.

A 14-year-old previously healthy girl was admitted to Infectious Disease Department of University Clinical Center of Kosova in Prishtina, complaining in headache, fever, right earache, and stiffness of the neck and right side torticollis. Diagnosis of meningitis was established according to the symptoms, physical exam, blood analyzes values, and results of lumbar puncture. Blood analyses were as follows: erythrocyte sedimentation rate (ESR) 80 mm/first hour, white blood count (WBC) 22,000 per square mm, and C-reactive protein (CRP) value was 126. Lumbar puncture results showed about 200 cells (leucocytes)/square mm, and manitol 20%, dexamethasone, analgesics-antipyretic, and antibiotics (ceftriaxone and vancomycine) were administered i.v. immediately. Consultation of ENT specialist, 2 days later, revealed right side AOM, with tenderness in mastoid tip palpation. The neck in the right posterior triangle was swollen and tender in palpation. Myringotomy under general anesthesia was performed, and suppurative content is released from middle ear cavity. A swab for bacteriology exam was obtained for culture, and the result, 2 days later, revealed *Streptococcus pneumoniae* as a causative agent. A CT scan, performed two days later, showed right side mastoid cells filled with liquid and thickening of mucosa. Besides this, a collection of possible suppurative content was detected in posterior triangle of right side of the neck, near the mastoid tip (Bezold's abscess). No imaging signs for intracranial spreading of ear infection were detected (Figure 1). The patient was transferred to our department (ENT), and an urgent operation was performed by the first author on the same day: cortical mastoidectomy with neck abscess drainage. The mastoid cells were found to be filled with thick mucosa and suppuration. All mastoid cells were cleaned, and *aditus ad antrum* was widened. A myringotomy procedure was performed, and a ventilation tube was inserted. Bezold's abscess was evacuated from the mastoid side and from neck incision. All mastoid cells from the sinodural angle (Citelli's angle) were cleaned; the bone above sigmoid sinus was thinned and did not show any infiltration of sinus. The bone cover of the lateral sinus was without pathologic changes. A drainage tube was put from the neck incision. The mastoid cavity was tamponed with gauze imbibed in local antibiotic/cortisone and retro auricular incision was not sutured. Metronidazole is added in postoperative medical therapy. Neck drainage from Bezold's abscess was washed out with syringe with saline and antibiotic (gentamicin) twice a day for the next 10 days, and the mastoid tamponade was changed every second day under general anesthesia. The patient showed excellent improvement clinically. Incision was sutured and drainage was removed. Laboratory analyses returned to normal values. Repeated lumbar punctures gave normal result. An MRI of the head was performed two weeks after the operation, and it showed a total thrombosis of lateral (sigmoid) sinus and partial thrombosis of transverse sinus of the right side. An anticoagulant therapy was given for 14 days subcutaneously. After therapy, the control MRI with contrast (magnetic resonance venography, MRV) showed the same picture: total thrombosis of lateral sinus and partial thrombosis of the transverse sinus of the right side with healing of the neck and mastoid cavity (Figure 2). In 30 months of follow-up, the patient has definitive asymptomatic LST and dry normal hearing ear.

## 3. Discussion

The use of antibiotics in treatment of acute otitis media (AOM) reduced the incidence of the acute mastoiditis (AM). During 1950s, 0.4% of episodes of AOM developed into AM, whereas the incidence of AM during 1980s dropped to 0.004% [12]. However, mastoiditis still occurs, and intracranial and extracranial complications can occur if AM is not managed in time. In Britain, only six cases of lateral sinus thrombosis and one case of Bezold's abscess have been reported in the literature in the last decade of the 20th century [13]. In our only tertiary medical center in Kosovo, in a 17-year period (1994–2011), the first author reported 2 cases of BA and 18 cases of LST as complications of cholesteatoma, but none from AM [14, 15]. This presented case is the first reported case of concomitant BA and LST arising from acute mastoiditis.

In all reported cases of BA/LST, the clinical appearance was typical. Earache, tender palpation in the mastoid tip, and neck stiffness and torticollis with high body temperature and clinical appearance of transitory meningitis are first symptoms and signs to raise suspicion on severe complication. If the body temperature changes periodically, the so-called septic temperature, this must warn clinicians of the possible thrombophlebitis, with danger of development of the fatal sepsis [3, 4, 8]. The patient we present in this paper had the all symptoms and signs mentioned above.

The acute mastoiditis is caused by the aerobe or anaerobe bacteria. Au et al. report in a review of 104 cases of pediatric LST from literature that from swabs taken during surgery or during suppuration, in 46% of cases, a single bacterial organism was isolated, with beta hemolytic *Streptococcus*, *Streptococcus pneumoniae*, and *Staphylococcus aureus* being the most common [10]. In our presented case, we took the swab during myringotomy, and the bacteriology result revealed *Streptococcus pneumonia* as the causative agent, and the ordinate antimicrobial therapy was culture based.

In the diagnostic of BA and LST, besides symptoms and signs, laboratory analyses and bacteriology examination, the imaging techniques are mainstay and represent the golden standard. In the cases with BA, on CT imaging, there is usually unilateral opacification of the middle ear and the mastoid cavities, often associated with bone erosion, especially of the mastoid tip. The collection of pus can be detected along the SCM muscle [7]. There are also reports of use of the ultrasound on detection of purulent contents in the neck. MRI and MRV are more sensitive in LST diagnosis and can be used for follow-up of the thrombosis [4, 8–10]. During the management of this particular case, with the help of CT imaging, we diagnosed and treated in time the acute mastoiditis and BA. The diagnosis of LST was made later during postoperative treatment, and the discussion of managing team were in the issue of origin of thrombosis. In the end, we agreed that the cause of thrombosis was the pressure of pus collection on the internal jugular vein (IJV) that subsequently caused the LST. This conclusion was made based on the findings during mastoidectomy, when we found normal bone covering the lateral sinus. Further search on literature must be conducted to conclude or exclude this statement.

Therapy of complications of the acute mastoiditis is medical, and antibiotics are chosen on the basis of the bacteriology exam, empirically and surgically, depending on the complications. All experts who care for the patients with mastoiditis and most of the publications on this topic advocate the administration of i.v. antibiotics and eventually ventilation tube in first 48 hours after the hospitalization and repeat the analyzes. If there is clinical improvement and normalization of analyzes, antibiotics continue for next 5 to 10 days. If there is no improvement, mastoidectomy has to be performed. In case of the complications, especially in the case of multiple complications, surgical treatment must be performed urgently. In our reported case, we first did a ventilation tube insertion and then mastoidectomy and BA evacuation and drainage. The LST was developed in the course of disease, caused from the pressure of pus collection in the neck. This causes the stopping of the blood flow through the venous sinus and causes consecutive thrombosis.

As a conclusion, early detection of developing complication of AM is the cornerstone of effective treatment of these complications. Multiple complications, although rare, still can threaten life of young patients.

## Abbreviations

AM: Acute mastoiditis
AOM: Acute otitis media
BA: Bezold's abscess
CCOM: Cholesteatomatous chronic otitis media
CRP: C-reactive protein
CT: Computed tomography
EEC: Extracranial complication
ENT: Ear, nose, throat
ESR: Erythrocyte sedimentation rate
ICC: Intracranial complication
LST: Lateral sinus thrombosis
MRI: Magnetic resonance imaging
MRV: Magnetic resonance venography
WBC: White blood count.
